# Retrieving hidden atrial repolarization waves from standard surface ECGs

**DOI:** 10.1186/s12938-018-0576-3

**Published:** 2018-11-06

**Authors:** Wei-Hua Tang, Wen-Hsien Ho, Yenming J. Chen

**Affiliations:** 1 Division of Cardiology, Department of Internal Medicine, National Yang-Ming University Hospital, Yilan, Taiwan; 2 Department of Healthcare Administration and Medical Informatics, Kaohsiung Medical University, 100 Shin-Chuan 1st Road, Kaohsiung, 807 Taiwan; 3 Department of Medical Research, Kaohsiung Medical University Hospital, Kaohsiung, Taiwan; 4 Department of Logistics Management, National Kaohsiung University of Science and Technology, 1 University Road, Yenchao, Kaohsiung, 824 Taiwan

**Keywords:** Atrial repolarization waves, Reaction–diffusion system, Electrophysiology model, Kalman filter, Monte Carlo Markov chain

## Abstract

**Background:**

This study estimates atrial repolarization activities (*Ta* waves), which are typically hidden most of the time from body surface electrocardiography when diagnosing cardiovascular diseases. The morphology of *Ta* waves has been proven to be an important marker for the early sign of inferior injury, such as acute atrial infarction, or arrhythmia, such as atrial fibrillation. However, *Ta* waves are usually unseen except during conduction system malfunction, such as long QT interval or atrioventricular block. Therefore, justifying heart diseases based on atrial repolarization becomes impossible in sinus rhythm.

**Methods:**

We obtain TMPs in the atrial part of the myocardium which reflects the correct excitation sequence starting from the atrium to the end of the apex.

**Results:**

The resulting TMP shows the hidden atrial part of ECG waves.

**Conclusions:**

This extraction makes many diseases, such as acute atrial infarction or arrhythmia, become easily diagnosed.

## Background

Atrial repolarization waves (*Ta* waves) are equally important as ventricular repolarization waves (*T* waves) and can exhibit significant potential as an effective biomarker for clinic diagnosis [1, 2]. However, depolarization is stronger than repolarization, and ventricular activities are even considerably stronger than atrial activities. To separate relatively weak activities in atria, existing studies have invested significant efforts in this aspect to help in myocardial diagnosis for either sinus rhythms and arrhythmia [3–8]. In the literature, no study has yet retrieved atrial repolarization activities because these activities can only be normally observed in patients under certain special ectopic pacing [2].

In this study, we develop a method to reveal hidden *Ta* waves in the majority of patients. Unlike in the direct processing of electrocardiogram (ECG) signals, an independent cycle of atria electrical activities is obtained by extracting the atrial part of transmembrane potentials (TMPs) of whole-heart activities and mapping partial TMPs back onto the ECG of a body surface. Despite the simple back-forward process, obtaining *Ta* waves with acceptable quality is challenging in computational design because the accuracy of inverse transformation highly relies on the fidelity of organ geometries and conductivities [9, 10]. Constructing a 3D mesh model for each patient can be expensive, and thus, our solution for the inverse problem assumes that all the physical measures of an individual are unreliable, and these parameters should be re-estimated during computation. The algorithm only requires long observation periods to perform self-adaptive and reliable statistical inferences for unsure parameters.

The morphologies of *P* waves and hidden *Ta* waves are typically considered important among healthcare professionals. In [1] and [11], *Ta* waves were suggested to be an early sign of inferior injury, such as acute atrial infarction or arrhythmia. One example is atrial fibrillation if the waves can be observed during conduction system malfunction, such as long QT interval or atrioventricular block.

*P* waves alone are important in justifying heart diseases in both sinus rhythm and fibrillation [12]. However, *P* waves are relatively weak, and thus, current understanding on atria activities are generally insufficient. Few studies have investigated activities from the atria part of myocytes. In [13], the problem was initially addressed via computer simulation. Without involving inverse problems, [13] facilitated a forward model, which mapped current dipoles onto atrial mid-myocardium to surface ECGs under a set of predetermined parameters to understand the contributions of right and left atria activities to the observed *P* waves during sinus rhythm and atrial fibrillation.

In clinical situations, *Ta* waves are not only hidden, but *P* waves are also tangled with the adjacent QRS complex. In [7] and [8], statistical and signal processing methods were, respectively used to single out *P* waves from the rest of the QRS part. Surface signals are complex combinations of current stimuli from millions of cardiomyocytes; thus, the signal separation task must be performed at the level of myocardium cells, and solving an ill-posed inverse problem is inevitable. Pioneer source models, such as those in [14–16], have been integrated into and advanced for contemporary computational electrocardiology models that establish models for various processes ranging from cellular bioelectrical activities to body surface potential distribution [17–19]. The forward problems involve mapping from inter- to intra-cellular currents onto body surface potential distributions [20, 21]. Given the nature of complexity in the biological field, the large degree of freedom poses a challenge in evaluating inverse problems.

When dealing with the inverse problem, most regulation methods can only condition numerical difficulty from the mathematical point of view; however, the problem of multiple solutions must be addressed by restoring possible missing constraints. In [22], this difficulty was solved by reducing the inverse problem in limited mapping from epicardial to body surfaces. In [23], the inverse problem was addressed by proposing a special equipment that could collect thousands of potentials on the body surface instead of the standard 12-lead ECG. Activation time sequencing or imaging can be evaluated for primitive diagnosis without retrieving detailed cellular activities [24–28]. However, electric current constraints from the ionic behavior of individual cells, such as in [17], impose additional computational challenge given the millions of myocytes. Moreover, additional constraints introduce other unknown parameters, and the degree of freedom remains high. Therefore, advanced statistical methods should be applied to obtain reliable solutions.

Existing studies continuously contribute to addressing the problem of physiological models, and specific body surface electrical data from patients are always being corrupted by noise and the incorrect construction of organ geometries. In [29], spatial covariance in a volume conductor was facilitated for maximum a posteriori (MAP) equation. In [30], temporal and spatial covariances were estimated under certain mathematical assumptions based on structures that were inherent in the space–time correlation matrix. In [31], the facilitation of multiple information sources to improve the efficiency of Bayesian MAP formulation was suggested. In [32], TMPs were constrained using a diffusion–reaction model from cellular activation dynamics, which limited the inverse problem in both spatial and temporal dimensions. This work suggested relying on a statistical method to address both model and data errors in terms of prior knowledge on cell current dynamics and evidence for surface potential data. In [33], the progress of statistical identification from the perspective of systems biology was reviewed.

## Methods

As mentioned earlier, the extraction of *P* waves should be conducted at the electric current level in myocardial sources. The model for the cardiac computational system comprises two parts according to the component guideline in [34]. The first part involves mapping between body surface potentials and intra-cellular TMPs. Evaluating TMPs is considered a difficult inverse problem given a potential map of a body surface [35, 36]. The second part aims to constrain the inverse problem, in which the constraint describes changes in TMPs in terms of electrical propagation between myocardia. Most electrophysiological models are diffusion–reaction systems [17, 36–38].

### Inverse problem

We first consider the forward problem from equivalent current–dipole sources to body surface potentials. The sources of bioelectric currents across cell membranes excite the movement of cardiomyocytes and induce potential fields, which can be detected via surface electrodes. The total current density is presented as $\varvec{J}(\varvec{r}) = \varvec{J}_{s} (\varvec{r}) + \sigma \varvec{E}(\varvec{r})$, where $\varvec{J}_{s}$ is the net source current density ($A/m^{2}$); $\sigma$ is conductivity in homogenous dielectric media; and $\varvec{E}$ is the electric field, which exhibits the relation $\varvec{E} = - \nabla \varPhi$ for potential function $\varPhi (\varvec{r})$. Vector fields are denoted as bold face symbols, such as current density $\varvec{J}(\varvec{r})$, which is a vector field at location $\varvec{r}$. The total current $\nabla \cdot \varvec{J} = 0$ diverges without external current under quasi-static conditions. Thus, $\nabla \cdot (\sigma \nabla \varPhi ) = \nabla \cdot \varvec{J}_{s}$, and the relation between measured potentials and heart sources is transformed into a Poisson equation. For cardiac volume $V_{H}$, the potentials are primitively expressed as $\varPhi (\varvec{r}) = \frac{1}{4\pi \sigma }\iiint_{{V_{H} }} {\varvec{J}_{s} (\varvec{r^{\prime}}) \cdot \nabla \left( {\frac{1}{{|\varvec{r} - \varvec{r^{\prime}}|}}} \right)d^{3} \varvec{r^{\prime}}}$.

To model equivalent current density, the entire myocardium is divided into grid meshes. Following the suggestion in [39], boundary element methods are applied. The potential $\varPhi$ at the body surface is maintained as $\varPhi$, and TMP is denoted as $\varvec{u}$. By tessellating and vectorizing all cardiac and thorax surfaces, a discrete matrix Eq. (1) is obtained as suggested in [32] and [40].

1 $$\phi (t) = \varvec{Lu}(t),$$

where $\varvec{L}$ is the discretized transfer matrix that converts TMP $\varvec{u}$ to surface potential $\phi_{8}$. When the vectorized body surface potentials are only sampled at eight electrode positions for the standard 12-lead ECG signals, the potentials are denoted as $\varPhi_{8}$ for clarity.

The transfer matrix $\varvec{L}$ is synthesized with the geometries and conductivities of the organs inside the thorax. The geometric coordinates are segmented and discretized via magnetic resonance imaging (MRI) or computed tomography for a specific patient. Given numerical sensitivity and unavoidable movement, the forward model may suffer from geometric errors and should be incorporated as a part of modeling [9, 41]. In [42], geometric errors were suggested to be overcome by using Bayesian MAP estimation or Kalman filtering with Gaussian geometric errors. In the present study, we do not rely on the accuracy of geometry and conductivity. We estimate the parameters along with the process of estimating TMPs [43, 44]. Bayesian estimation in error covariance enables performance analysis to statistically characterize solutions.

### Reaction–diffusion systems

Electrical propagation between myocardia is typically modeled differently in terms of complexity level—from the simplest Eikonal model at the tissue level, through bidomain/monodomain models and phenomenological models, to the most complicated ionic models at the cellular level. Phenomenological models focus at the macroscopic level and ranges from 2-variable equations [14, 37] to the complicated 15-variable Luo–Rudy model [45]. Resolution is not a concern in extracting *P* waves. Electrical propagation is captured using the reaction–diffusion system [37] with the same setting as that in [46, 47]. Considering the balance between precision and computation, a simple system is sufficient to constrain the ill-posed inverse problem. Therefore, we adopt the system from [37] as follows:

2 $$\left\{ {\begin{array}{*{20}l} {\frac{{\partial \varvec{u}}}{\partial t} = (\nabla (\varvec{D}\nabla \varvec{u}) + k\varvec{u},\varvec{u} - \varvec{a},1 - \varvec{u} - < \varvec{u},\varvec{v} > )} \hfill \\ {\frac{{\partial \varvec{v}}}{\partial t} = - e(\varvec{v} + k < \varvec{u},\varvec{u} - \varvec{a} - 1 > } \hfill \\ \end{array} } \right.,$$

where $\varvec{u}$ and $\varvec{v}$ are the column vectors of TMPs and recovery current, respectively; and the operator $< , >$ represents a component-wise multiplication. $D$ is the diffusion tensor; and $k$, $a$, and $e$ are the parameters. By converting the equation into finite element meshes [47], the reaction–diffusion system can then be used as an effective constraint in solving the inverse problem. Let $\varvec{x} = [\varvec{u},\,\varvec{v}]$. The system can then be written as $\dot{\varvec{x}} = F_{d} (\varvec{x})$, where $F_{d} (\varvec{x}) = \left[ {\left( {\nabla \left( {\varvec{D}\nabla \varvec{u}} \right) + k\varvec{u},\varvec{u} - \varvec{a},1 - \varvec{u} - < \varvec{u},\varvec{v} > } \right)\;and \; - e(\varvec{v} + k < \varvec{u},\varvec{u} - \varvec{a} - 1 > } \right]$.

### Hierarchical estimation

Our problem contains a large number of uncertainties, and thus, advanced Bayesian statistics can be a viable approach [44]. The basic idea is to estimate the posterior probability of the unknown cardiac source $P(\varvec{x}_{k} |\phi_{1:k} )$ based on an a priori distribution of the sources $P(\varvec{x})$ and a group of affecting parameters. When (1) and (2) are combined, we obtain the data model as follows (3):

3 $$\left\{ {\begin{array}{*{20}l} {\dot{\varvec{x}}_{k + 1} } \hfill & = \hfill & {F_{d} (\varvec{x}_{k} ) + \varvec{w}_{k} ,} \hfill \\ {\phi_{k} } \hfill & = \hfill & {\varvec{Hx}_{k} + \varvec{z}_{k} ,} \hfill \\ \end{array} } \right.$$

where $\varvec{H} = [\varvec{L} + \Delta \varvec{L}\;;\;0]$ is the output matrix with uncertainty $\Delta \varvec{L}$, and $\varvec{w}$ and $\varvec{z}$ are two i.i.d. error processes with zero means and covariances $\varvec{\xi}_{w}$ and $\varvec{\xi}_{z}$. Given that the model does not rely on the accuracy of the heart and torso geometries, the error terms in the elements of the transfer matrix $L$ are embedded into the matrix with random variables $\Delta \varvec{L}$. Let $\theta = (k,a,e)$ to incorporate the parameters in the reaction–diffusion function $F_{d} ( \cdot )$. Therefore, the parameters for the process comprise $\Delta \varvec{L}$ and $\theta = (k,a,e)$.

The recursive estimation for the posterior probability density $P(\varvec{x}_{k} |\phi_{1:k} )$ can be conceptually achieved in two steps. The forecast term $P(\varvec{x}_{k} |\phi_{1:k - 1} )$ can be obtained through Chapman–Kolmogorov integration $\mathop \smallint \nolimits P(\varvec{x}_{k} |\varvec{x}_{k - 1} )P(\varvec{x}_{k - 1} |\phi_{1:k - 1} )d\varvec{x}_{k - 1}$, given that the posterior $P(\varvec{x}_{k - 1} |\phi_{1:k - 1} )$ is known from time $k - 1$, and $P(\varvec{x}_{k} |\varvec{x}_{k - 1} )$ is determined from the system equation. The current time posterior $P(\varvec{x}_{k} |\phi_{1:k} )$ is updated using the Bayes rule $\frac{{P\left( {\phi_{k} |\varvec{x}_{k} } \right)P\left( {\varvec{x}_{k} |\phi_{1:k - 1} } \right)}}{{P\left( {\phi_{k} |\phi_{1:k - 1} } \right)}}$, where $P(\phi_{k} |\phi_{1:k - 1} ) = \mathop \smallint \nolimits P(\phi_{k} |\varvec{x}_{k} )P(\varvec{x}_{k} |\phi_{1:k - 1} )d\varvec{x}_{k}$.

To deal with a large number of parameters, the guideline in [46] and [47] indicates that the complicated joint distribution in data model (3) can be formulated as a hierarchical model and factorized into a series of conditional distributions. The guideline suggests that the random variables to be estimated can be factored into three stages, such that $p({\text{process}},{\text{parameters}}|{\text{data}}) \propto$ $p({\text{data}}|{\text{process}},{\text{parameters}})$ $p({\text{process}}|{\text{parameters}})$ $p({\text{parameters}})$. Therefore, the joint posterior distribution can be written in a hierarchical form as follows:

4 $$P(\varvec{x},\Delta \varvec{L},\theta ,\varvec{\xi}_{w} ,\varvec{\xi}_{z} |\phi ) \propto P(\phi |\varvec{x},\Delta \varvec{L},\varvec{\xi}_{z} )P(\varvec{x}|\theta ,\varvec{\xi}_{w} )P(\Delta \varvec{L})P(\varvec{\xi}_{z} )p(\theta )P(\varvec{\xi}_{w} ).$$

Following the suggestion in [47], a Monte Carlo Markov chain (MCMC) slice sampler [48] is applied in the Bayesian computation model because of the high dimension in our complex problem. A full Bayesian analysis of this problem is achieved by sampling the joint posterior distribution (13) using an MCMC technique called slice sampling [49]. Another potential solution for reducing the constraining effects of prior knowledge is the simultaneous estimation of the TMP dynamics and electrophysiological properties of the myocardium. This method has the advantage that the constraining models can be modified according to the collected data of patients with filtering of unknown parameters.

### Experiment setup

**Fig. 1:**
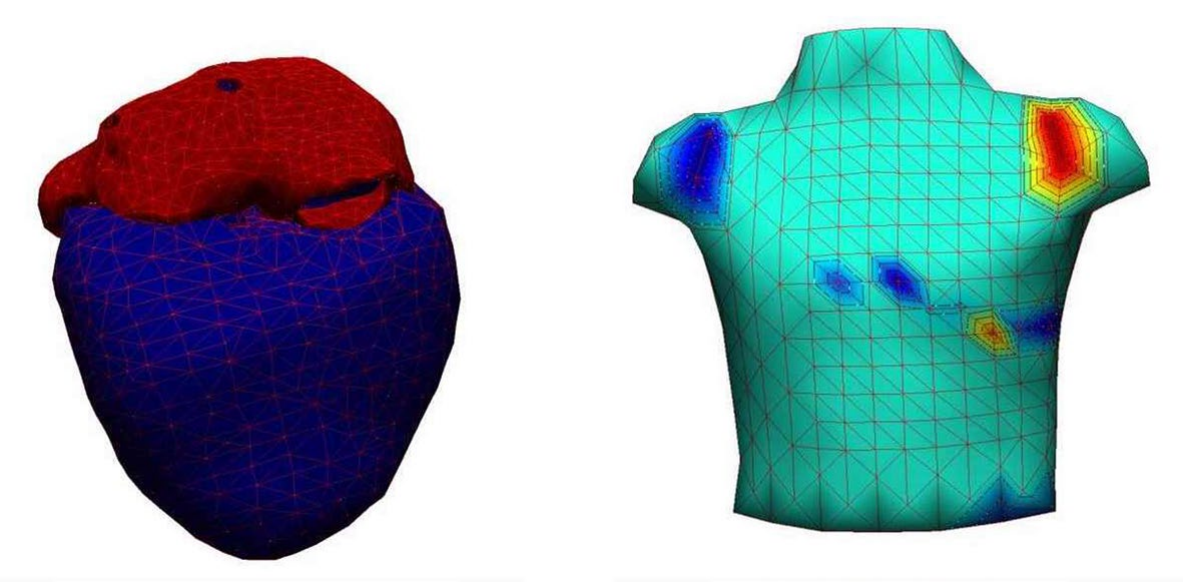
Geometries of heart and torso

To conduct the following experiments, 3D geometric models of a complete heart and torso are necessary. Cardiac geometric data were adopted from the ECGSim data set, which described a healthy normal young male using complete atria and ventricles (Fig. 1, with 1634 nodes for atria and 1500 nodes for ventricles) [50]. Given that a 3D imaging will not be constructed on the epicardial surface, the requirement for grid size is low. Resolution is further reduced to prevent the introduction of excessive numerical difficulties from the source of the standard 12-lead ECG.

The geometry of a torso was adopted from the PhysioNet data archive, which also originated from the body surface mapping data of Dalhousie University [51–53]. Although accuracy is not a concern, mapping between surface nodes to the electrode positions of standard leads should be specified. Given the well-prepared recording and documentation in the data set, the detailed mapping from the surface nodes to the 15 standard leads was elaborated.

The ECG data were also adopted from PhysioNet: ptbdb and incartdb [51]. The signals were preprocessed to eliminate electromagnetic interference, baseline wandering (e.g., electromyographic noise), and various artifacts (e.g., electrode motion) [54].

The implementation programs for the experiments were developed in MATLAB and R. The transfer matrix was produced using the open source SCIRun/BioPSE from the Scientific Computing and Imaging Institute of the University of Utah [18, 55].

**Fig. 2:**
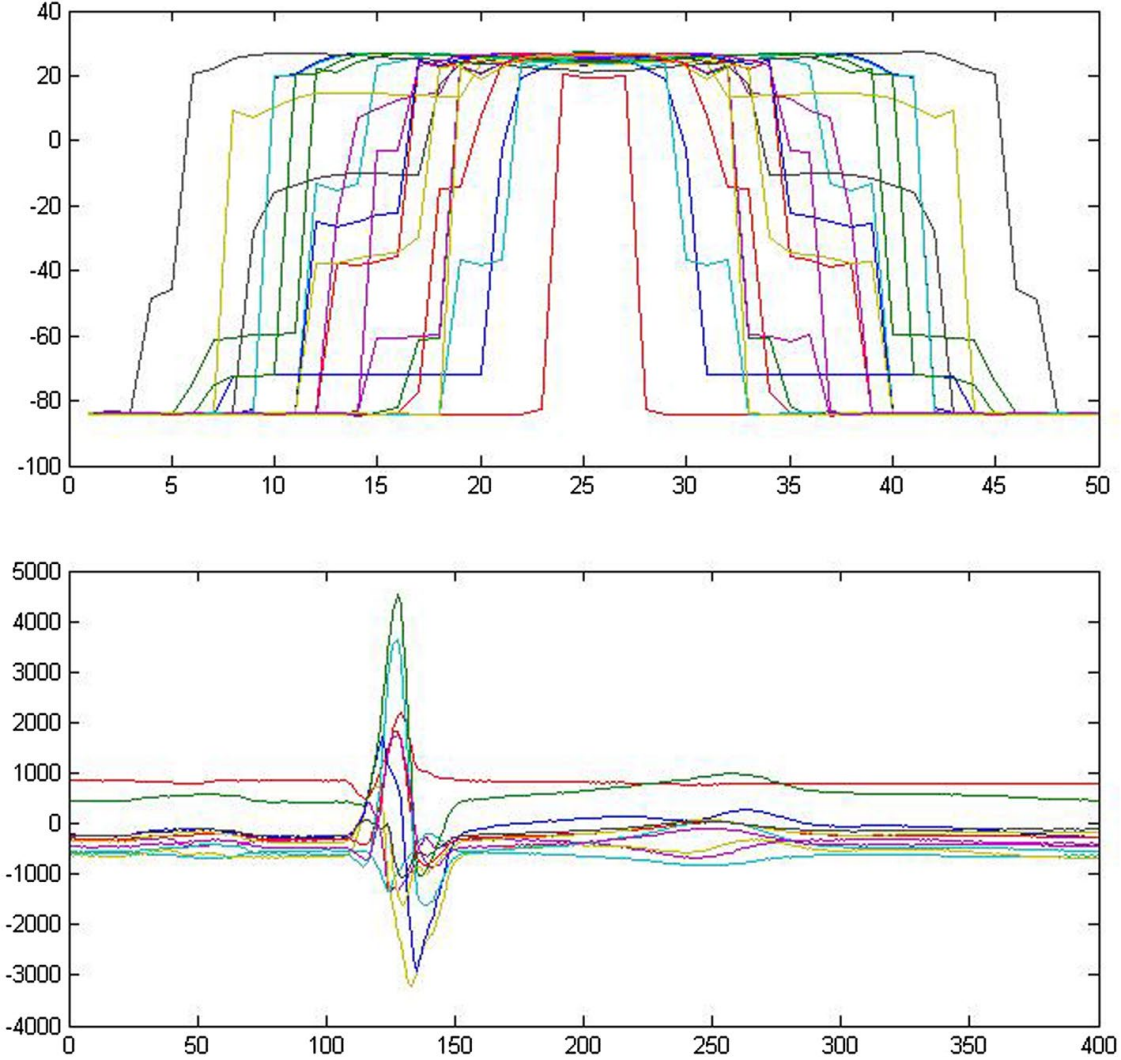
TMP and surface ECG

This study develops a model that retrieves hidden atrial repolarization waves by solving an inverse problem from surface ECG to cardiac TMPs (Fig. 2), where an ill-posed problem is constrained by temporal and spatial electrophysio-relations. The modeling approach can only be maintained at a coarse level because the source data are limited by the number of channels in the standard lead ECG. By contrast, cardiac electrical signals can be estimated by being modeled as a stochastic process with unknown excitation parameters and continuous acquisition of signals. In the solving process, several issues are encountered and need to discuss further.

**Fig. 3:**
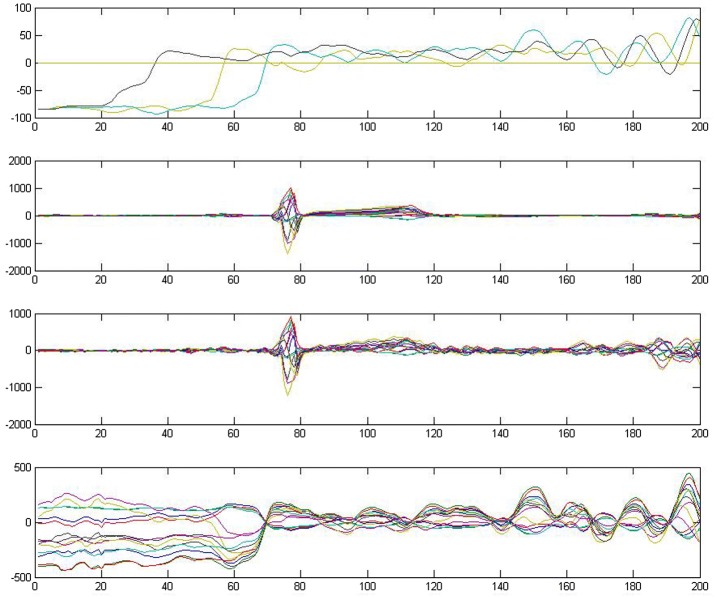
Results of 12-lead ECG with MCMC. Top: atrial part of TMP; 2nd: original ECG; 3rd: simulated ECG; bottom: atrial part of simulated ECG

The experiment presents good results. As shown in Fig. 3, the top panel presents the inverse solution for TMPs in the atrial part of the myocardium. The figure reflects the correct excitation sequence starting from the atrium to the end of the apex. When we multiply the entire TMPs to the transfer matrix, the forward problem restores the original ECG, as shown in the third panel. The figure exhibits good approximation of the original ECG (second panel), except for several ripples near the end of the cycle. This result is considered good because the resolution is under 14 nodes on the body surface and 20 nodes in the myocardium. The bottom panel shows the extracted atrial electric activities. Each line in the graph corresponds to one of the 14 nodes that constitute the standard 12-lead ECG.

## Discussions

The error caused by spatial digitization may overwhelm other sources of error terms because of the resolution limitation in mesh grids. The errors caused by modeling, geometric, and measuring uncertainties are integrated into a reaction–diffusion system, which is a stochastic state-space system with a powerful capability to estimate true values among noise background. The two-variable reaction–diffusion system (2) is linearized for convenient computation experiments. All the parameters are embedded into each individual matrix element, which will be estimated in the subsequent MCMC step. Although sophisticated and accurate models for cardiac electrophysiological dynamics can be used, the estimation results may not improve in our case.

One of the error sources originates from the inhomogeneity and anisotropy of conductivity among different torso tissues. To obtain an acceptable imaging of the heart surface, the anisotropy of the intracellular conductivity tensor along the fiber structure of myocardia must be considered. In this study, we adopt a real-time estimation approach and leave all the parameters to be estimated during the online training step.

As mentioned earlier, given the lack of spatial resolution, we do not expect the complete accuracy of the inverse and forward simulations. Although an ordinary Moore–Penrose pseudo-inverse can obtain a perfectly low root-mean-square error after performing backward and forward projections, the cardiac potential maps obtained from this pseudo-inverse are meaningless. Therefore, the computation objective is to reconstruct a TMP distribution that closely mimics a real TMP distribution [17]. We determine that the sequence and heterogeneity of TMPs throughout the myocardium significantly influence the final waveform of surface ECG.

The selection of prior distributions and initial conditions can influence the final results of the inverse solution when the Bayesian approach is used. During our MCMC estimation step, we perform our experiments by assuming that the noise and error terms are Gaussian distributed. Accordingly, we also select Gaussian as their conjugate prior for assigning prior distribution. The Gaussian assumption is reasonable for the coarse computation level. If further investigation is conducted, then non-Gaussian or empirical distribution can be used to estimate unknown parameters under the hierarchical Bayesian estimation framework.

By contrast, initial conditions are considerably sensitive to the algorithm. If parameters or matrices are available in published studies, then we will use their well-known values as initial amounts. Patient-specific variation should be accommodated by the random sampling process. A good initial condition can make convergence fast and accurate. In certain situations, convergence may not reach each selected initial value. For example, numerical difficulty may occur for a certain prior distribution. Several procedures can improve numerical stability, including increasing the condition numbers of covariance matrices. However, their details are considered state-of-art and require further elaboration.

For computational convenience, the reaction–diffusion system (2) is linearized into a matrix equation because matrix operations are more efficient than the *n*th order Runge–Kutta methods with nonlinear cross-terms. However, the order of the system will increase significantly from nonlinear to linear. We artificially augment a large number of co-state variables in system (2); that is, we add ten times more dummy variables following variable $\varvec{v}$. The number of dummy variables significantly influences the quality of TMP reconstruction.

## Conclusions

In this study, we consider only signals in the standard 12-lead ECG measurement. Unlike most studies, our approach relies on neither accurate geometries nor high-resolution body surface potential maps. We only use a rough model as an initial guess and then simultaneously estimate the inverse solutions and model parameters. In this setup, we eliminate the requirement to construct a geometric model of the heart using off-line MRI pictures. Our method simultaneously estimates inverse potential solutions and geometric details.

We separate the atrial part of ECG waves from the rest of the signals based on a standard clinical ECG recording. Traditional approaches [7, 8] work only on signal morphology and attempt to cancel out the large QRS-T complex from several standard templates. We proceed directly through TMPs in the myocardial cells and extract the electricity generated by atria only. The obtained surface ECG contains only *P* waves and is more robust than those obtained via waveform approach are.

Future development can focus on improving prior distribution specifications and initial conditions. We will additionally construct empirical distribution for all error terms to be a basis of the prior distribution. The algorithm will be continuously improved and parallelized for better computational efficiency.
